# Use of Mid-Upper Arm Circumference Band in Wasting Detection in Children with Cerebral Palsy in Türkiye

**DOI:** 10.3390/children12081002

**Published:** 2025-07-30

**Authors:** Uğur Topçu, Çiğdem Lazoğlu, Caner Aslan, Abdurrahman Zarif Güney, Zübeyr Kavcar, Orhan Coşkun

**Affiliations:** 1 Department of Pediatrics, Gaziosmanpasa Training and Research Hospital, University of Health Sciences, Istanbul 34255, Türkiye; caner.aslan@saglik.gov.tr (C.A.); zubeyr.kavcar@saglik.gov.tr (Z.K.); 2 Department of Actuarial Sciences, Hacettepe University, Ankara 06100, Türkiye; cigdemkobal@hacettepe.edu.tr; 3 Cerrahpasa Faculty of Medicine, Istanbul University-Cerrahpasa, Istanbul 34098, Türkiye; abdurrahman.guney@iuc.edu.tr; 4 Department of Pediatric Neurology, Gaziosmanpasa Training and Research Hospital, University of Health Sciences, Istanbul 34255, Türkiye; orhan.coskun5@saglik.gov.tr

**Keywords:** cerebral palsy, malnutrition, mid-upper arm circumference (MUAC), wasting

## Abstract

**Background/Objectives**: Malnutrition is a common problem in children with cerebral palsy (CP). The aim of this study was to investigate the suitability and diagnostic performance of mid-upper arm circumference (MUAC) z-score in diagnosing wasting in children with CP, and its impact on diagnostic accuracy when evaluated concomitantly with additional clinical factors (birth weight, history of phototherapy). **Methods**: This single-center, cross-sectional study included 83 children with CP, aged 6 months–17 years, followed-up in our clinic. Anthropometric measurements (MUAC, Body Mass Index (BMI)) and clinical data (birth weight, history of phototherapy, Gross Motor Function Classification System (GMFCS)) were prospectively collected. Wasting was defined according to the BMI z-score ≤ −2 criteria. The diagnostic performance of MUAC z-score was evaluated by Receiver Operating Characteristic (ROC) analysis. The contribution of additional covariates was examined using logistic regression analysis and the backward elimination method. **Results**: MUAC z-score alone demonstrated good discrimination in diagnosing wasting with an Area Under the Curve (AUC) value between 0.805 and 0.821, but its sensitivity was limited (67.0%). No statistically significant difference was found in diagnostic performance between MUAC measurements of the right arm, left arm, and the unaffected arm (*p* > 0.050). In logistic regression analysis, MUAC z-score (*p* = 0.001), birth weight (*p* = 0.014), and a history of phototherapy (*p* = 0.046) were found to be significantly associated with wasting malnutrition. The simplified model including these variables yielded an AUC value of 0.876. **Conclusions**: MUAC z-score is a usable tool for wasting malnutrition screening in children with CP. Although its sensitivity is limited when used alone, its diagnostic accuracy increases when evaluated concomitantly with additional clinical factors such as birth weight and a history of phototherapy. This combined approach may offer clinicians a more robust tool for the early diagnosis and management of wasting malnutrition in children with CP.

## 1. Introduction

Cerebral palsy (CP) is a lifelong neurodevelopmental disorder that emerges in early childhood, affecting motor functions and posture, which is non-progressive but can change over time. In addition to motor impairment, sensory, perceptual, cognitive, behavioral, and communication problems, epilepsy, and secondary musculoskeletal problems to the disease frequently accompany CP [1].

Due to deficiencies in motor functions, feeding, digestion, and swallowing processes can also be affected in children diagnosed with CP; consequently, the risk of malnutrition increases [2]. The most commonly used anthropometric indicators in the diagnosis of malnutrition are weight-for-length (WFL < 24 months), weight-for-age (WFA ≥ 24 months), Body Mass Index (BMI; >2 years), and mid-upper arm circumference (MUAC; 2 months–18 years) [3].

Nutritional assessment in children with CP is complicated by significant challenges in obtaining reliable anthropometric data. A key difficulty is the accurate measurement of linear height due to secondary motor impairments such as joint contractures, spasticity, spinal deformities like scoliosis and positioning problems which are prevalent in this population [4,5]. These physical limitations can render standard height-dependent indicators, such as Body Mass Index (BMI), inaccurate or impossible to obtain.

Similarly, while obtaining weight is generally more feasible, achieving a precise and reliable reading can be compromised by abnormal muscle tone and involuntary movements that interfere with stable positioning on a scale [2,5]. Gross Motor Function Classification System (GMFCS) indicates a child’s level of gross motor function and mobility, allowing ranking from I (light symptoms) to V (more severe) [5]. A large percentage of children with CP, particularly those with higher GMFCS levels, cannot stand independently. This makes the use of a standard stand-on scale impossible and immediately necessitates alternative methods. These challenges with both height and weight measurements underscore the need for practical and reliable alternative measures. In the last few decades, several methods to determine anthropometrics in children with CP have been developed, but with no consensus reached on the ideal method despite extensive discussion on the topic [6]. One of the methods, MUAC, measured in millimeters, has the following main advantages: It provides the degree of malnutrition directly as a z-score with a single-step measurement. It is inexpensive, rapid, and independent of age and sex. Also, it does not require training and can be easily applied by healthcare personnel [7].

Nevertheless, even if patients are assessed with appropriate anthropometric data, primarily MUAC, the absence or non-use of appropriate growth charts specific to children with CP can lead to assessment errors [5,8]. Specific charts for children with CP are available, such as Brooks charts [9]. These charts reflect how children with CP have grown, rather than how they can be expected to optimally grow [10]. Therefore, while our study investigated the suitability of MUAC z-score measurement and how its diagnostic performance changes in the assessment of patients with CP, it also aimed to develop a new perspective in predicting wasting using other data obtainable alongside MUAC.

Against MUAC’s beneficial skills, the existing literature offers limited evidence on the diagnostic accuracy of MUAC for detecting wasting in children with CP. Therefore, the hypothesis emerges that the sensitivity of MUAC as a standalone screening tool may be insufficient, and that additional factors should be considered to enhance its diagnostic power. In this context, the potential contribution of readily available clinical data from patient records to the diagnostic performance of MUAC is a critical and underinvestigated area. If this easily obtainable data can augment the diagnostic power of MUAC, clinicians would have a more robust and practical screening tool to detect wasting early in children with CP. Therefore, this study aims to investigate the diagnostic performance of the MUAC z-score alone and also to determine its impact on diagnostic accuracy when evaluated concomitantly with additional clinical factors, such as birth weight and a history of phototherapy, in children with CP.

## 2. Materials and Methods

This single-center, prospective study was conducted at Gaziosmanpasa Training and Research Hospital’s Pediatric Neurology Clinic, located in Istanbul, Türkiye. A total of 86 regularly followed children with cerebral palsy were selected who agreed to participate in the study between June 2023 and June 2024. A total of 3 patients with incomplete records for key anthropometric data (body weight, height, and MUAC) were excluded from the study. The study was approved by the Istanbul Gaziosmanpasa Training and Research Hospital Ethics Committee (Approval No.: 63) and was conducted in accordance with the principles of the Declaration of Helsinki.

Data for each patient were collected from the cerebral palsy patients, as well as from their families. The collected data included demographic information (age, sex), clinical characteristics (GMFCS level), perinatal history (birth weight, history of neonatal intensive care unit admission, history of phototherapy), and anthropometric measurements.

Body weight was measured using a digital physician’s scale, and height was measured with a stadiometer. MUAC (right arm, in the middle of arm) was measured with MUAC z-score tape. To ensure consistency, the same equipment was used for all participants. Also, for each participant, key measurements such as MUAC and height were taken twice by the same physician. If the two measurements differed by more than a pre-specified limit, a third measurement was performed. The average of the two closest readings was used for the final analysis, a procedure designed to minimize random measurement error. Body Mass Index (BMI) was calculated from each child’s height and weight, and BMI z-scores were determined using reference data for Turkish children (which account for both age and sex) provided by Olcay Neyzi et al. [11]. The presence of wasting malnutrition was defined, as the reference standard, by a BMI z-score ≤ −2.

### Statistical Analysis

Statistical analyses were conducted using R 4.2.2. To summarize sample characteristics, continuous variables were presented as mean ± standard deviation, while categorical variables were reported as frequency (percentage).

To evaluate the ability of MUAC z-scores to discriminate wasting malnutrition, Receiver Operating Characteristic (ROC) curve analysis was performed. This is a standard method used to assess the diagnostic performance of continuous predictors. The Area Under the Curve (AUC) was used as a measure of performance. To compare AUC values across different measurement approaches, the DeLong test was applied, as it allows statistically valid comparisons between correlated ROC curves.

Logistic regression analysis was conducted to identify independent predictors of malnutrition. This method was chosen due to the binary nature of the outcome variable (presence or absence of malnutrition) and its ability to simultaneously adjust for multiple covariates. To improve model simplicity and avoid overfitting, a backward elimination strategy was employed, which iteratively removed non-significant variables based on predefined significance thresholds (*p* > 0.050). The final model’s performance was assessed using AUC and the Akaike Information Criterion (AIC), where lower AIC values indicate better model fit.

## 3. Results

### 3.1. Study Population Characteristics

A total of 83 children with CP were included in this prospective, single-center study. The descriptive and clinical characteristics of the participants are summarized in Table 1 and Table 2. The mean age of the children was 7.9 years, with a range of 6 months to 17 years. The sample consisted of 43 (52.0%) male and 40 (48.0%) female participants.

The cohort presented a diverse spectrum of motor impairment according to the Gross Motor Function Classification System (GMFCS), with the following distribution: GMFCS level I (*n* = 28, 33.9%), level II (*n* = 27, 32.5%), level III (*n* = 14, 16.8%), and levels IV-V combined (*n* = 14, 16.8%). Based on the reference standard of a Body Mass Index (BMI) z-score ≤ −2, wasting malnutrition was identified in 25.0% (*n* = 21) of the children.

### 3.2. Diagnostic Performance of MUAC Z-Score Alone

The analyses showed that MUAC z-score alone has a good discriminatory power for detecting wasting in individuals with CP, with an Area Under the Curve (AUC) value between 0.805 and 0.821. However, the sensitivity at the optimal cut-off point was 67.0%, and the Positive Predictive Value (PPV) was in the range of 53.0–58.0%.

**Figure 1:**
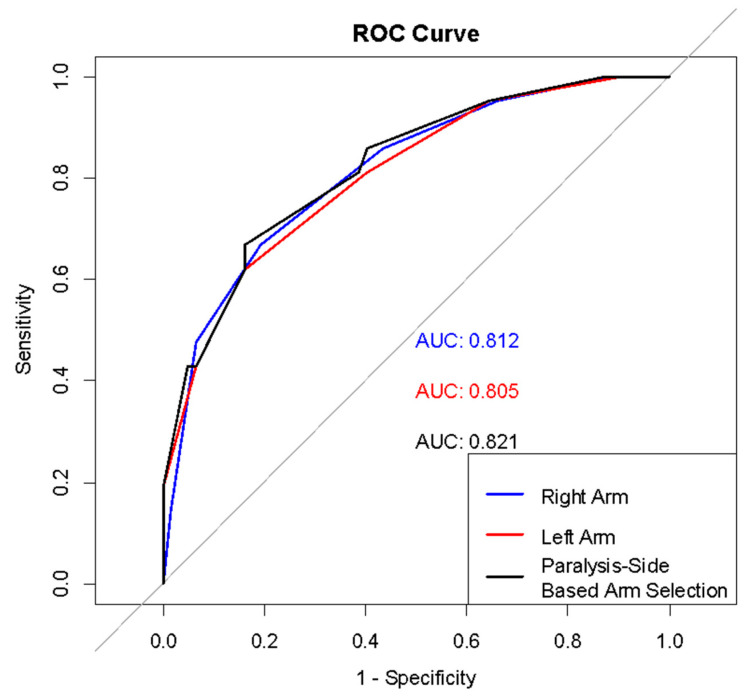
ROC curves for arm measurements in wasting detection.

In the ROC analysis comparing measurements from different limbs, no statistically significant difference was found between the AUC values for the right arm, left arm, and the arm determined according to paralysis status (*p* > 0.050) (Figure 1), (Table 3 and Table 4).

### 3.3. Improvement of Diagnostic Performance with Logistic Regression Model

To identify the most significant determinants of wasting, a logistic regression model was developed and optimized using the backward elimination method. After this process, birth weight (*p* = 0.014), mean MUAC z-score (*p* = 0.001), and a history of phototherapy (*p* = 0.046) remained as the statistically significant variables in the final model. Age, sex, history of neonatal intensive care unit (NICU) admission, and GMFCS status were excluded from the model as they were not found to be significant predictors. The model’s goodness of fit improved, as indicated by a decrease in the AIC from 74.586 in the initial model to 69.738 in the final, simplified model (Table 5 and Table 6).

**Figure 2:**
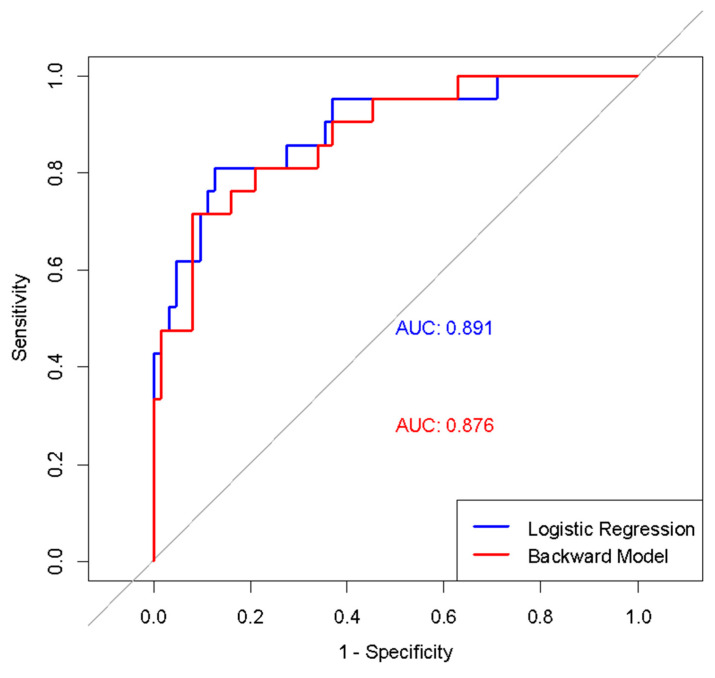
ROC curves of initial and simplified logistic regression models in diagnosing wasting.

The diagnostic performance of the models was compared using AUC values. The initial comprehensive model had an AUC of 0.891, while the final simplified model had a strong AUC of 0.876. Both regression models demonstrated higher discriminatory power compared to using the MUAC z-score alone (Figure 2).

## 4. Discussion

It is estimated that approximately 17 million children worldwide live with a diagnosis of CP, and a significant portion of them are at high risk for malnutrition [12]. Our study investigated the role and diagnostic performance of the MUAC z-score in children with wasting malnutrition.

Our primary findings reveal that while MUAC as a standalone tool has good discriminatory power, this performance is characterized by high sensitivity and low specificity. Crucially, our study demonstrates that the diagnostic accuracy is significantly enhanced when the MUAC z-score is combined with key perinatal factors, namely birth weight and a history of phototherapy, within a logistic regression model. The performance profile of MUAC is critical for its clinical application. Its high sensitivity suggests that it is a valuable screening tool for wasting in the CP population. A normal MUAC measurement makes underlying wasting (as defined by BMI) highly unlikely, allowing clinicians to confidently rule out risk in many children quickly and non-invasively. However, the limited specificity is a significant drawback for its use as a standalone diagnostic tool. This low specificity results in a high number of false positives, meaning it may incorrectly label normal children as being at risk. This finding aligns with the literature that calls for caution when applying single anthropometric measures to neurologically impaired children, whose body compositions differ significantly from their healthy peers [13,14]. Also, it is suggested that MUAC has low reliability in diagnosing severe acute malnutrition in infants ≤ 6 months [15]; however, there were no cases under 6 months in our study.

From a practical standpoint, our finding that there was no statistically significant difference in diagnostic performance when measuring the right, left, or unaffected arm offers important guidance. This provides clinicians with valuable flexibility, simplifying the measurement process in children with contractures or severe spasticity where accessing a specific limb may be difficult. The European Society for Paediatric Gastroenterology, Hepatology and Nutrition (ESPGHAN) guidelines do not specify an opinion on specific arm selection in the nutritional assessment of neurologically impaired children so our findings align with the guidelines [16].

When contextualizing our findings within the existing literature, the 25.0% wasting prevalence in our cohort presents an interesting comparison. A study from Türkiye including 1057 neurologically impaired children (15.8% with CP) found the overall malnutrition rate to be 17.1%, while this rate was higher at 36.0% in patients with CP [17]. In another study examining 68 neurologically impaired children aged 1–17 years (83.8% with CP), when anthropometric measurements (weight, height, triceps skinfold thickness (TSFT), MUAC) were evaluated as z-scores, malnutrition was detected in 49.5% of the patients [18]. This difference may be attributable to variations in the distribution of GMFCS levels or inclusion of other types of malnutrition. While authors note that the prevalence of malnutrition is high or low, especially in children with severe or light degree of gross motor function impairment (GMFCS level) [19,20], all of the patients in our study (*n* = 14) with GMFCS levels IV and V had wasting in accordance with the level.

The most novel contribution of our study is the demonstration that a combined model significantly outperforms MUAC alone (AUC 0.876). The inclusion of low birth weight and a history of phototherapy as significant predictors is particularly noteworthy. The significance of low birth weight in our model is biologically reasonable, as it is a well-established risk factor for subsequent growth failure and morbidity in the CP population [21]. Similarly, while a history of phototherapy is not a direct cause of malnutrition, it often serves as a proxy for significant perinatal stress, such as prematurity or severe hyperbilirubinemia. These conditions are known to be associated with a higher risk of adverse neurodevelopmental outcomes [22]. Therefore, our model suggests that these markers of perinatal vulnerability, when combined with a direct measure of current nutritional status like MUAC, create a more powerful and holistic predictive tool. This highlights that the risk of malnutrition in CP is not only defined by the child’s present condition but is also deeply rooted in their perinatal history. To the best of our knowledge, this is the first study to identify these specific perinatal risk factors as significant predictors within such a model for children with CP.

This finding expands on previous research, which recommended combining MUAC with age and GMFCS level [19], by introducing easily obtainable historical data that adds powerful predictive value.

This study has several strengths, including its novel approach of creating a predictive model combining anthropometric and perinatal data and the direct comparison of different arm measurement techniques. However, we acknowledge its limitations. The single-center and the limited sample size may affect the generalizability of our findings. Furthermore, a broader challenge in the field, which also applies to our study, is the use of standard z-scores. As you noted, current specific growth charts for children with CP are controversial, and the high prevalence of malnutrition in this population makes it difficult to establish “normal” reference values. Therefore, using adapted targets from healthy population data, as was performed in this study, remains a reasonable approach until validated, CP-specific curves are developed. Another limitation of our study is its specific focus on wasting malnutrition, which meant we did not include a concurrent analysis of chronic malnutrition, or stunting, as indicated by height-for-age z-scores. Stunting is a highly prevalent and clinically significant issue in the CP population, often reflecting long-term nutritional deficits and impacting overall growth potential. By focusing solely on wasting (as indicated by BMI), our study does not provide a comprehensive overview of the full spectrum of malnutrition present in our cohort. This focused approach was intentional, as our primary objective was to evaluate the performance of MUAC as a screening tool for acute malnutrition, for which BMI-for-age is the most appropriate indicator across our wide age range. Therefore, future prospective studies are needed to simultaneously evaluate indicators of both acute and chronic malnutrition in this population. Understanding the relationship between MUAC, BMI, and height-for-age would provide a more holistic view of the nutritional challenges faced by children with CP. Also, the sample size did not allow for a stratified analysis by age group. Future studies with larger cohorts should investigate whether the diagnostic performance of MUAC differs between infants, children, and adolescents with CP.

## 5. Conclusions

In conclusion, our findings support that MUAC is a practical screening tool in the clinical follow-up of children with CP; however, this study demonstrated that it is insufficient for diagnostic accuracy when used alone.

The primary finding of our research is that the diagnostic power of MUAC increases significantly when combined with easily accessible perinatal risk factors, such as low birth weight and a history of phototherapy. This integrated model offers clinicians a more practical and powerful tool to identify the risk of wasting in children with CP earlier and more accurately, ultimately contributing to the timely planning of appropriate nutritional interventions.

## Figures and Tables

**Table 1:** Clinical characteristics of the study population (N = 83).

Variable	Category	*n* (%)
Nutritional Product	Using Medical Formula	29 (35.0%)
	Not Using Medical Formula	54 (65.0%)
Gender	Male	43 (52.0%)
	Female	40 (48.0%)
Admission to NICU	Yes	22 (26.5%)
	No	61 (73.5%)
History of Phototherapy	Yes	19 (23.0%)
	No	64 (77.0%)
Wasting Status (Based on BMI)	Yes	21 (75.0%)
	No	62 (25.0%)
MUAC Score (Upper Right Arm)	≤−4	4 (5.0%)
	(−4)–(−3)	10 (12.0%)
	(−3)–(−2)	12 (14.5%)
	(−2)–(−1)	19 (23.0%)
	(−1)–(0)	16 (19.0%)
	(0)−(1)	15 (18.0%)
	(1)−(2)	5 (6.0%)
	(2)−(3)	2 (2.5%)
Type of Motor Deficit	GMFCS Stage I	28 (33.9%)
	GMFCS Stage II	27 (32.5%)
	GMFCS Stage III	14 (16.8%)
	GMFCS Stage IV–V	14 (16.8%)

**Table 2:** Descriptive characteristics of the study population.

Variables	Mean	Standard Deviation
Gestational Age at Birth (weeks)	34.02	±4.85
Age (months)	94.65	±50.48
Birth Weight (kilograms)	2.29	±1.04

**Table 3:** Diagnostic accuracy metrics based on arm measurement and paralysis side.

Arm Used	True Positive	False Positive	True Negative	False Negative	Positive Predictive Value	Negative Predictive Value	Kappa
Right	14	12	50	7	53.8%	87.7%	0.44
Left	13	10	52	8	56.6%	86.6%	0.44
Dominant/Paralyzed Side	14	10	52	7	58.8%	88.1%	0.48

**Table 4:** ROC analysis results (“95% CI”, 2000 stratified bootstrap replicates).

Arm Used	Sensitivity	Specificity	95% CI	Best Threshold
Right	0.67	0.81	0.71 to 0.92	−2.00
Left	0.67	0.81	0.70 to 0.91	−2.00
Dominant/Paralyzed Side	0.67	0.81	0.72 to 0.92	−1.75

**Table 5:** Logistic regression results before and after backward elimination.

Variable	Logistic Regression		Backward Model	
	Estimate	*p*-Value	Estimate	*p*-Value
Intercept	−0.67	0.693	−1.04	0.236
Nutritional Product	1.18	0.121	1.23	0.072
Gender	−1.27	0.127	−	−
Birth Weight (kg)	−0.84	0.060	−0.94	0.014
Admission to NICU	1.09	0.264	−	−
Phototherapy	−2.68	0.019	−1.98	0.046
Age	−	0.568	−	−
MUAC Score	−1.38	<0.001	−1.21	0.001
Type of Motor Deficit	−0.09	0.868	—	−

**Table 6:** Performance comparison of logistic regression models before and after backward elimination.

Model Criteria	Logistic Regression	Backward Model
Null Deviance	93.893 (df=82)	93.893 (df=82)
Residual Deviance	56.586 (df=74)	59.738 (df=78)
Akaike Information Criterion (AIC)	74.586	69.738
Degree of Freedom (df)	74	78
Fisher Scoring Iterations	6	6

## Data Availability

The data presented in this study are available on request from the corresponding author due to privacy reasons.

## Abbreviations

The following abbreviations are used in this manuscript:

MUAC	Mid-Upper Arm Circumference
BMI	Body Mass Index
CP	Cerebral Palsy
WFL	Weight-For-Length
WFA	Weight-For-Age
NICU	Neonatal Intensive Care Unit
AIC	Akaike Information Criterion
AUC	Area Under the Curve
GMFCS	Gross Motor Function Classification System
TSFT	Triceps Skinfold Thickness
ESPGHAN	European Society for Paediatric Gastroenterology, Hepatology and Nutrition
